# Realm of PD-(D/E)XK nuclease superfamily revisited: detection of novel families with modified transitive meta profile searches

**DOI:** 10.1186/1472-6807-7-40

**Published:** 2007-06-20

**Authors:** Lukasz Knizewski, Lisa N Kinch, Nick V Grishin, Leszek Rychlewski, Krzysztof Ginalski

**Affiliations:** 1 Interdisciplinary Centre for Mathematical and Computational Modelling, Warsaw University, Pawinskiego 5a, 02-106 Warsaw, Poland; 2 Howard Hughes Medical Institute and Department of Biochemistry, University of Texas Southwestern Medical Center, 5323 Harry Hines Blvd, Dallas, TX 75390-9050, USA; 3 BioInfoBank Institute, Limanowskiego 24a, 60-744 Poznan, Poland

## Abstract

**Background:**

PD-(D/E)XK nucleases constitute a large and highly diverse superfamily of enzymes that display little sequence similarity despite retaining a common core fold and a few critical active site residues. This makes identification of new PD-(D/E)XK nuclease families a challenging task as they usually escape detection with standard sequence-based methods. We developed a modified transitive meta profile search approach and to consider the structural diversity of PD-(D/E)XK nuclease fold more thoroughly we analyzed also lower than threshold Meta-BASIC hits to select potentially correct predictions placed among unreliable or incorrect ones.

**Results:**

Application of a modified transitive Meta-BASIC searches on updated PFAM families and PDB structures resulted in detection of five new PD-(D/E)XK nuclease families encompassing hundreds of so far uncharacterized and poorly annotated proteins. These include four families catalogued in PFAM database as domains of unknown function (DUF506, DUF524, DUF1626 and DUF1703) and YhgA-like family of putative transposases. Three of these families represent extremely distant homologs (DUF506, DUF524, and YhgA-like), while two are newly defined in updated database (DUF1626 and DUF1703). In addition, we also confidently identified an extended AAA-ATPase domain in the N-terminal region of DUF1703 family proteins.

**Conclusion:**

Obtained results suggest that detailed analysis of below threshold Meta-BASIC hits may push limits further for distant homology detection in the 'midnight zone' of homology. All identified families conserve the core evolutionary fold, secondary structure and hydrophobic patterns common to existing PD-(D/E)XK nucleases and maintain critical active site motifs that contribute to nucleic acid cleavage. Further experimental investigations should address the predicted activity and clarify potential substrates providing further insight into detailed biological role of these newly detected nucleases.

## Background

Restriction endonuclease-like proteins, also called a PD-(D/E)XK nucleases, constitute a large and diverse superfamily of enzymes that are involved in numerous nucleic acid cleavage events important for various cellular processes. The SCOP [1] database currently groups 23 families of known structure in the restriction endonuclease-like superfamily, including among others 15 different restriction endonucleases [2], holiday junction resolvases (endonuclease I, Hjc) [3,4], lambda exonuclease [5] and very short patch repair (Vsr) endonuclease [6]. Their function varies from repairing damaged DNA (Vsr), resolving holliday junctions (endonuclease I, Hjc), performing additional cleavage events in DNA recombination (lambda exonuclease), to protection of host organisms against foreign DNA invasion (restriction endonucleases).

Despite displaying very little sequence similarity, the restriction endonuclease-like enzymes retain a common core fold that consists of a central four-stranded mixed β-sheet flanked by an α-helix on either side (with αβββαβ topology). The general architecture of the restriction endonuclease-like fold allows recognition of diverse nucleic acid substrates, which may vary from specific palindromic DNA sequences (type II restriction endonuclease and Vsr) to unique DNA backbone structures (Hjc). The substrate specificity in many cases arises from various insertions to the core fold that may even encompass entire domains [7,8].

The restriction endonuclease-like superfamily possesses a relatively conserved active site PD-(D/E)XK signature (motif II and motif III), critical for cleaving the nucleic acid phosphodiester bond [9-11]. The signature lysine residue is responsible for positioning water for in-line attack on the substrate phosphodiester bond, while the carboxylates coordinate up to three metal ions that serve as cofactors in the reaction. Several variations to this named motif exist, and additional family conserved charged or polar residues located in core helices of the fold (motif I and motif IV) contribute to active site architecture in many cases [11].

Standard homology detection methods usually fail to recognize novel PD-(D/E)XK nucleases due to lack of significant sequence similarity between families and numerous insertions to the core fold. In previous work we applied a transitive search approach using the meta profile comparison method Meta-BASIC and identified nine new restriction endonuclease-like fold families among hypothetical proteins [12]. Some of these families were also detected by others using HHsearch method [13]. In this study we employed a modified transitive search procedure by including additional below threshold score Meta-BASIC hits in updated PFAM and PDB databases and identified five more novel PD-(D/E)XK nuclease families.

## Results and discussion

**Figure 1:**
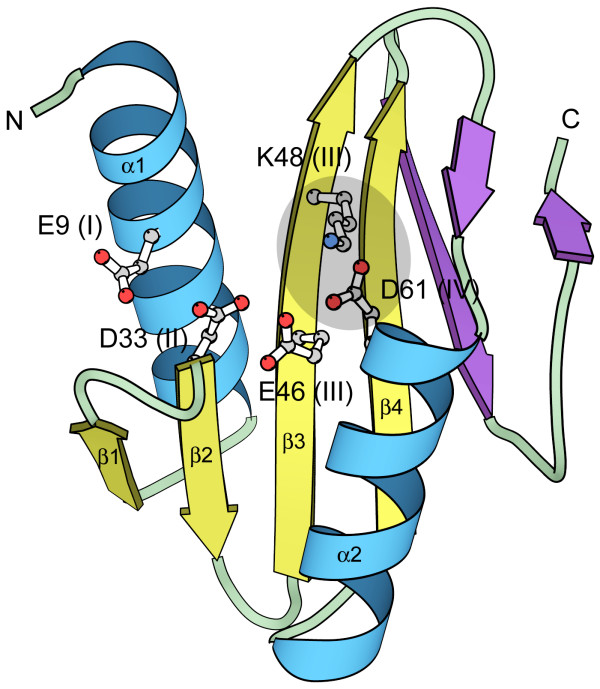
**Typical PD-(D/E)XK nuclease structure**. Ribbon representation for archaeal holliday junction resolvase (Hjc, pdb:1gef) with the critical motif I (E9), motif II (D33), motif III (E46, K48) and motif IV (D61) active site residues shown as balls-and-sticks. Secondary structure elements of the conserved core fold are labeled and colored blue (α-helix) and yellow (β-strand), while additional insertions to the core are colored violet. In several detected families, conserved motif III lysine migrates to motif IV where (frequently mutated to arginine) it may occupy a similar spatial position (highlighted in grey) and thus play an equivalent functional role.

**Figure 2:**
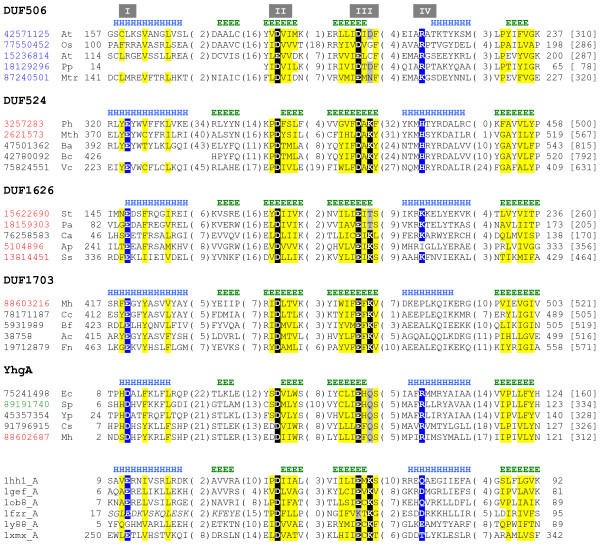
**Novel PD-(D/E)XK nuclease families**. Multiple sequence alignment of representative sequences from newly detected families and selected PD-(D/E)XK nuclease structures for the conserved structural core. Sequences are labeled according to gi number or PDB code and an abbreviation of the species name: Ac *Azotobacter chroococcum*, Ap *Aeropyrum pernix*, At *Arabidopsis thaliana*, Ba *Bacillus anthracis*, Bc *Bacillus cereus*, Bf *Bacteroides fragilis*, Ca *Chloroflexus aurantiacus*, Cs *Chromohalobacter salexigens*, Ct *Clostridium tetani*, Ec *Escherichia coli*, Fn *Fusobacterium nucleatum*, Mh *Methanospirillum hungatei*, Mtr *Medicago truncatula*, Mth *Methanothermobacter thermautotrophicus*, Os *Oryza sativa*, Pa *Pyrobaculum aerophilum*, Ph *Pyrococcus horikoshii*, Pp *Pinus pinaster*, Sp *Sodalis phage*, Ss *Sulfolobus solfataricus*, St *Sulfolobus tokodaii*, Vc *Vibrio cholerae*, Yp *Yersinia pesti*. Sequence gi numbers are colored according to taxonomy: with bacterial in black, archeal in red, eukaryotic in blue and viral in green. The first and last residues numbers are indicated before and after each sequence with total sequence length following in square bracket. The numbers of excluded residues are specified in parentheses. Residue conservation is denoted with following scheme: uncharged, highlighted in yellow; charged or polar highlighted in grey; small, letters in red. Active site PD-(D/E)XK signature residues are highlighted in black while other conserved polar/charged residues in alternative active site positions are highlighted in blue. Restriction endonuclease-like motifs (I-IV) are labeled on the top. Predicted (gi:42571125, gi:3257283, gi:15622690, gi:88603216 and gi:75241498) and observed (pdb:1hh1) secondary structure elements (E, β-strand; H, α-helix) are indicated above the sequences. Italicized sequence corresponds to domain-swapped region of 1fzr.

This study is a continuation of our previous work identifying novel restriction endonuclease-like fold families among catalogued PFAM families (mainly domains of unknown function, DUFs) using transitive searches with the Meta-BASIC method. Although existing restriction endonuclease-like structures retain similar active site residues within the same core fold (with αβββαβ topology) (Figure 1), they exhibit extreme structural diversity (structure comparison scores can be below threshold [12]). Since Meta-Basic scores are benchmarked using rigorous structural criteria to define confidence thresholds (predictions with Z-score above 12 have <5% probability of being incorrect [14]), the structural diversity of PD-(D/E)XK nuclease fold can be reflected as lower than threshold Meta-BASIC scores. Accordingly, we modified our previous transitive search approach to consider potentially correct Meta-BASIC predictions placed (according to Z-score) among unreliable or incorrect ones. While this method extension provides an effective strategy for detecting extremely distant homologs, the resulting non-trivial predictions require additional criteria for justification. Therefore, we demand that family profiles of low-scoring hits retain all core secondary structure elements comprising the nuclease fold and conserve critical functional residues essential for function. Confirmation of these predictions by a consensus of fold recognition methods, such as those produced by 3D-Jury must also support the non-trivial links. Consequently, an extensive search of the updated GRDB database, which stores Meta-BASIC connections between PFAM families and PDB structures, led to identification of five new putative PD-(D/E)XK nuclease families (not detectable with standard sequence search methods) encompassing hundreds of so far uncharacterized proteins of unknown function. Three of these families represent extremely distant homologs (DUF506, DUF524, and YhgA-like), while two are newly defined in the updated GRDB system (DUF1626 and DUF1703). In addition to conserving critical features of the restriction endonuclease-like fold, all families display similar hydrophobicity patterns to known PD-(D/E)XK nucleases (Figure 2). All predictions are discussed in details below.

### DUF506

DUF506 family (PF04720) embraces a number of plant proteins including 18 copies from *Oryza sativa*, 23 copies from *Arabidopsis thaliana *and single sequences from *Pinus pinaster *and *Medicago truncatula*. Meta-BASIC mapped the consensus sequence of DUF506 onto holliday junction resolvase (Hjc) structure (pdb:1hh1[15], Meta-BASIC score 8.2) and PFAM family UPF0102 (PF02021, Meta-BASIC score 7.6), which has been predicted as distantly related to Hjc. According to our benchmarks, predictions with Z-score >8 have <10% probability of being incorrect. In addition, several fold recognition servers also selected restriction endonuclease-like fold among first 5 models. DUF506 proteins possess a predicted conserved core ααααβββαβαα secondary structure pattern (with putative restriction endonuclease-like core elements underlined). DUF506 family members retain the characteristic motif II (xDxxx motif located in the second core β-strand, where x is in general any hydrophobic residue) of the PD-(D/E)XK signature, with the exception of three conservative replacements of aspartate for glutamate in gi:15236814, gi:18399441 and gi:20147393. Motif III is represented by a (D/E)X(D/N/S/C/G) pattern (Figure 2). The missing positively charged residue in motif III is possibly replaced by a conserved arginine in motif IV (R218 gi:42571125) located in the proceeding α-helix (Figure 1). A similar migration of this critical active site residue can be observed for the DUF790 family [12]. Although one of the family members, *Pinus pinaster *protein (gi:18129296), has been reported to be expressed in native cells [16], this prediction represents the only functional information available for this family of sequences. DUF506 proteins lack any identified fused domains that might hint at biological function, and detailed analysis of a genomic context did not help identify potential physiological roles for the family.

### DUF524

The DUF524 family (PF04411, COG1700) includes a number of hypothetical proteins of bacterial origin (in addition to two sequences from *Bacillus cereus *[17] with a fused McrB restriction GTPase domain that are annotated as 5-methylcytosine-specific restriction related enzymes) and two sequences from archeal species (*Pyrococcus horikoshii *and *Methanothermobacter thermautotrophicus*). The C-terminal region of DUF524 consensus sequence was mapped by Meta-BASIC onto the PD-(D/E)XK nuclease families DUF1064 (PF06356, Meta-BASIC Z-score 9.3) [12] and Type I restriction enzyme R protein N terminus (HSDR_N, PF04313, Meta-BASIC Z-score 8.8) [18]. Additional support for this prediction was obtained with 3D-Jury although with below threshold scores due to inconsistent alignments generated by servers.

DUF524 family proteins consist of at least two domains: a C-terminal PD-(D/E)XK nuclease domain and an N-terminal region of yet unknown function with a predicted all β secondary structure pattern followed by mainly α-helical structure. The DUF524 restriction endonuclease-like domain has two additional β-strands inserted to the core fold after the first core α-helix (αβββββαβ topology, conserved core elements are underlined). Similar insertion in this region can be found in BsoBI restriction endonuclease (pdb:1dc1) [19]. The PD-(D/E)XK signature is clearly conserved among DUF524 family members and corresponds to invariant PD (motif II) and DAK (motif III) motifs (Figure 2). Additionally, DUF524 proteins conserve a glutamic acid in motif I (E323 in gi:3257283), most likely involved in metal ion binding. Lastly, the second core α-helix contains an invariant MHXYRD motif (motif IV).

The COG corresponding to DUF524 (COG1700) has a confidently detected (STRING score 0.73) genomic neighbourhood association to a unique family of restriction endonuclease GTPase subunits (COG1401). These GTPases have been assigned to AAA+ class chaperonin-like ATPases [20] and include McrB of the *E. coli *methylation-dependent restriction system (McrBC). In this system, DNA cleavage by the McrC subunit is strictly coupled to GTP hydrolysis by the McrB subunit [21] instead of the typical ATP cofactor requirement of most restriction modification systems (for example Type I and Type III restriction endonucleases [22]. The McrC subunit responsible for cleavage is a PD-(D/E)XK endonuclease [21], which supports assignment of DUF524 to the restriction endonuclease-like superfamily and suggests a function of methylation-dependent restriction for this group of unknown proteins.

### DUF1626

DUF1626 family (PF07788, COG5493) includes 19 proteins from certain archeal (*Sulfolobus tokodaii*, *Sulfolobus solfataricus*, *Sulfolobus acidocaldarius*, *Pyrobaculum aerophilum*, *Thermofilum pendens *and *Aeropyrum pernix*) and bacterial (*Chloroflexus aurantiacus*, *Roseiflexus sp*., *Candidatus Kuenenia stuttgartiensis *and delta proteobacterium MLMS-1) organisms. The consensus sequence of this family was mapped with an above threshold scores to both PD-(D/E)XK PFAM families: DUF91 (PF01939, Meta-BASIC score 17.2) [12], restriction endonuclease (PF04471, Meta-BASIC score 14.9) and PDB structures: holliday junction resolvases Hje (pdb:1ob8[23], Meta-BASIC score 13.7) and Hjc (pdb:1hh1, Meta-BASIC score 12.1).

Majority of DUF1626 proteins possess an additional N-terminal α-helical region, mainly coiled-coil (as predicted with Coils2 [24]) and are frequently annotated as tropomyosin, coiled-coil or microtubule binding proteins. Specifically, RPS-BLAST searches of the Conserved Domain Database (CDD) [25] detect sequence similarity to several coiled-coil containing families, although the repeated sequence patterns found in coiled-coils render this similarity unreliable for any type of functional or evolutionary assumptions.

The DUF1626 restriction endonuclease-like domain has predicted αβββαβαβ topology (with conserved endonuclease elements underlined), where the C-terminal elements possibly extend the domain core. Motif III lysine of the PD-(D/E)XK nuclease fingerprint is often substituted by threonine (Figure 2) and it is likely that, similarly to DUF506 or DUF790 [12], the lysine migrated to an α-helix (motif IV) following the third core β-strand (Figure 1). Specifically, DUF1626 proteins with threonine in motif III possess a conserved patch of positively charged lysine and arginine residues in the motif IV α-helix that might be involved in substrate binding or contribute to active site formation.

### DUF1703

The DUF1703 family (NCOG44579) groups together a set of uncharacterized proteins from one archeal (*Methanospirillum hungatei*) and various bacterial organisms. The C-terminal region of DUF1703 consensus sequence has detectable similarity to PD-(D/E)XK nucleases (DUF91, above threshold Meta-BASIC score 16.7). Specifically, the DUF1703 C-terminal domain has the predicted secondary structure pattern of the restriction endonuclease-like fold core with an additional β-strand at C-terminus (αβββαββ, nuclease core underlined) and conserves all restriction endonuclease-like superfamily motifs (Figure 2). These include both PD-(D/E)XK signature motifs II and III as well as two other family conserve positions: a charged aspartate residue in the first core α-helix (E420 in gi:88603216, motif I), presumably responsible for metal ion binding, and a glutamine residue in the second core α-helix (Q480 in gi:88603216, motif IV), that may take part in binding of nucleic acid substrate.

**Figure 3:**
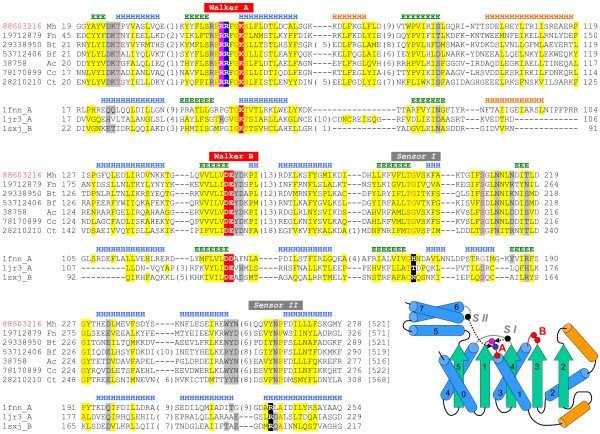
**N-terminal extended AAA-ATPase (AAA+) domain in DUF1703 family**. Multiple sequence alignment for the N-terminal region of representative sequences from DUF1703 family and selected AAA+ structures together with the schematic diagram of the fold. Sequences are labeled according to gi number or PDB code and an abbreviation of the species name: Ac *Azotobacter chroococcum*, Bf *Bacteroides fragilis*, Bt *Bacteroides thetaiotaomicron*, Cc *Chlorobium chlorochromatii*, Ct *Clostridium tetani*, Fn *Fusobacterium nucleatum*, Mh *Methanospirillum hungatei*. Sequence gi numbers are colored according to taxonomy: with bacterial in black and archeal in red. The first and last residues numbers are indicated before and after each sequence with total sequence length following in square bracket. The numbers of excluded residues are specified in parentheses. Residue conservation is denoted in following scheme: uncharged, highlighted in yellow; charged or polar highlighted in grey; small, letters in red. Invariant active site residues are highlighted in red, while additional active site residues that have migrated in DUF1703 family sequences from typical Sensor I and Sensor II positions (highlighted in black) are highlighted in pink and blue. AAA+ motifs (Walker A, Walker B, Sensor I and Sensor II) are labeled above corresponding residue columns. Locations of predicted (gi:88603216) and observed (pdb:1fnn) secondary structure elements (E, β-strand; H, α-helix) are marked above the sequences and are colored according to the schematic representation shown in the bottom right corner. Core AAA+ helices and strands are colored blue and green, respectively, while inserted helices are colored orange. Walker A and B motif loops are labeled and colored red, with invariant DUF1703 family residues that correspond to AAA+ active site residues depicted as red circles. Typical Sensor I and Sensor II sites are labeled and colored gray, with positions of functional sites depicted as black circles. Migrated DUF1703 residues (indicated by broken black arrows) that could substitute for Sensor I and Sensor II are denoted by pink and blue circles, respectively.

Meta-BASIC linked the DUF1703 N-terminal region to an archeal ATPase family (PF01637, above threshold Meta-BASIC score 20.6). The initial assignment was further supported with 3D-Jury that mapped this region with above threshold scores (>50) to several structures from the SCOP extended AAA-ATPases (AAA+) family of P-loop containing nucleoside triphosphate hydrolases fold (for instance pdb:1fnn[26], pdb:1sxj[27], pdb:1iqp[28], pdb:1jr3[29]). The DUF1703 AAA+ module consists of two domains: an N-terminal α/β domain (referred as domain 1) [20] with characteristic Walker A and Walker B motifs, and a small anti-parallel four-helix bundle domain (referred as domain 2). In typical AAA+ structures, residues from the Walker A motif (GXXGXGK(T/S)) and the Walker B motif (referred as DEAD or DEXX motif) together bind nucleoside and Mg^2+ ^in the deep cleft between two domains. While DUF1703 family proteins possess classical Walker B motif (DEYD), residues in the Walker A motif differ from the canonical definition: arginines replace the first and second glycines i.e. 44RPRRFGKS51 in gi:88603216 (described residues underlined) (Figure 3). Detailed analysis of AAA+ family sequences and structures revealed that similar changes to Walker A motif are possible (for instance GXX**R**XGKT in pdb:1v5w[30] and **L**XXSXGRS in pdb:1rif[31]), but they usually correlate with mutations in other positions in order to maintain active site pocket function, shape and accessibility. Other defined AAA+ family elements, such as the Sensor 1 and Sensor 2 regions [20] that help catalyze hydrolysis lack corresponding functional residues in DUF1703 sequences (Figure 3) have diverged in this family. Specifically, the Sensor 1 region (194FLTGKVS199 in gi:88603216), situated in a helical turn after strand 4 (Figure 3), conserves a glycine residue (G197 in gi:88603216) instead of a polar amino acid, while the Sensor 2 region (260GQQVYNP266 in gi:88603216), located at the N-termini of helix 7, lacks the typical arginine finger. We hypothesize that second and third conserved arginines in the DUF1703 family Walker A motif (44RPRRFGKS51 in gi:88603216) substitute for the Sensor I polar residue and the Sensor II arginine, respectively (Figure 3).

In summary, DUF1703 family proteins share a common four-domain structure, with an N-terminal AAA+ module (domains 1 and 2), a C-terminal restriction endonuclease-like domain and a small α-helical region (according to secondary structure predictions) in-between. Similar domain architecture can be found for other PD-(D/E)XK nucleases (for example, Mrr restriction endonuclease fused to NACHT ATPase domain in gi:68548712), providing further support for functional predictions.

### YhgA-like

The YhgA-like family (PF04754, COG5464) of putative transposases encompasses hundreds of bacterial proteins in addition to a few archeal (*Methanospirillum hungatei*) and one viral sequence from *Sodalis phage*. These proteins are assigned to the PD-(D/E)XK nuclease superfamily based on a detected Meta-BASIC connection to herpes virus protein UL24 (PF01646, Meta-BASIC Z-scores 11.6 for whole length consensus YhgA-like family sequence and above threshold 15.3 for selected N-terminal region encompassing putative restriction endonuclease-like domain), which has been recently identified as an additional member of this superfamily [32].

The predicted PD-(D/E)XK nuclease domain resides in the N-terminal region of YhgA-like proteins and displays a common predicted secondary structure αααβαββαβ pattern (putative restriction endonuclease-like core elements underlined), with two α-helical insertions to the core fold. Similar insertions of two α-helices before and a single α-helix after the first core β-strand can be found in RecB structure (pdb:1w36) [33]. In the YhgA-like family, the predicted restriction endonuclease-like domain is followed by a C-terminal α-helical region (~150 aa).

The YhgA-like family members clearly conserve active site motifs II and III (Figure 2), where motif III is identical to that of the Coi-A-like family (EXQ in the third core β-strand). Additional charged residues include an invariant motif I putative metal ion binding glutamate (D11 in gi:75241498) [8] and a motif IV arginine (R94 in gi:75241498). Importantly, a few bacterial sequences have glutamine substituted by lysine in motif III, nevertheless, glutamine is found in motif IV instead of conserved arginine. This evident sequence correlation in critical active site positions further stresses the correctness of the prediction for the YhgA-like family.

Using COG5464 (YhgA-like) as an input, the STRING database assigns a high confidence combined neighborhood and domain fusion score (0.755) to a group of uncharacterized cyanobacterial proteins (COG4636) that correspond to DUF820. DUF820 was previously identified as a PD-(D/E)XK nuclease (with a representative structure pdb:1wdj) [12], suggesting that the two families arose from a genetic duplication and may perform similar functions.

## Conclusion

The PFAM database currently defines a PD-(D/E)XK nuclease superfamily clan that groups 15 different families: including restriction endonuclease, archaeal holliday junction resolvase (Hjc), RmuC, herpes virus protein UL24, competence protein CoiA-like, sugar fermentation stimulation protein, VRR-NUC, herpesvirus alkaline exonuclease, DUF91, DUF790, DUF911, DUF1016, DUF1052, DUF1064 and UPF0102. Many of these families were identified in our previous studies [12,32]. In this work we performed systematic searches in updated PFAM database with transitive Meta-BASIC approach to further expand the realm of PD-(D/E)XK nuclease superfamily. We analyzed below threshold Meta-BASIC predictions to identify correct hits that due to their large evolutionary distance were assigned below cut-off confidence scores. Selection of these highly non trivial but reliable assignments was based on consistency of a predicted secondary structure pattern with that of the restriction endonuclease-like fold core, general conservation of hydrophobicity patterns, and presence of the signature PD-(D/E)XK nuclease motifs critical for function. This strategy resulted in identification of five new PD-(D/E)XK nuclease families in the PFAM database (DUF506, DUF524, DUF1626, DUF1703 and YhgA-like) encompassing hundreds of uncharacterized or hypothetical proteins. Additionally, analysis of genomic context for examined families strengthened several of our predictions. Altogether, obtained results suggest that combination of transitive Meta-BASIC searches with various other analyses (including sequence conservation, secondary structure prediction, domain architecture and genomic context) of below threshold hits may push limits further for distant homology detection in the 'midnight zone' of homology. Finally, using top-of-the line fold recognition methods we also identified AAA+ domain in the N-terminal region of DUF1703 proteins that is not detectable by standard sequence comparison methods.

## Methods

### Detection of putative PD-(D/E)XK nuclease families

Identification of novel PD-(D/E)XK nuclease families was carried out using GRDB system [14], which includes precalculated Meta-BASIC connections between PFAM (version 20.0) [34] families and proteins of known structure (PDB) [35]. Meta-BASIC is a consensus meta profile alignment method capable of finding very distant similarity between proteins through a comparison of sequence profiles enriched by predicted secondary structures (meta profiles).

We applied a similar transitive Meta-BASIC search approach as in our recent work [12], which identified a number of PD-(D/E)XK nuclease families exhibiting below threshold (<12) scores (according to rigorous structural criteria, scores above 12 have <5% probability of being incorrect [14]). Considering the structural divergence of restriction endonuclease-like fold [12] that is likely reflected as lower than threshold Meta-BASIC scores, we extended our previous transitive search method to consider the top 200 ranked Meta-BASIC hits for each query (including those hits that rank lower than the first false positive). Additionally, the steady increase in protein database sizes (PFAM, PDB and NCBI nr) may lead to straightforward detection of novel restriction endonuclease-like enzymes that were not detectable before or not included in previous versions of the GRDB system.

Using the collective set of previously identified PD-(D/E)XK nuclease families as queries, we applied transitive Meta-BASIC searches to an updated GRDB system. Selection of potentially correct, yet highly diverged families among the numerous low scoring Meta-BASIC predictions was based on two essential defining criteria for PD-(D/E)XK nucleases. First, family profiles must include correctly aligned conserved acidic residues from motifs II and III that contribute to cleavage. Second, family profiles must include all secondary structure elements (predicted by PSI-PRED [36]) that correspond to the evolutionary core of PD-(D/E)XK nuclease fold (αβββαβ). Consensus sequences and a few representative members of families that met the above criteria were submitted to the Meta Server [37] that assembles various secondary structure prediction and top-of-the-line fold recognition methods. Results produced by these diverse structure prediction methods were screened with 3D-Jury [38,39], a consensus approach that uses structural comparisons to select the best models (the most abundant structures) from a group of assembled models.

### Generation of multiple sequence-to-structure alignment

To collect protein sequences that belong to newly identified restriction endonuclease-like families, PSI-BLAST [40] searches (E-value threshold 0.001) were performed against the NCBI non-redundant (nr) protein database with the consensus sequences of analyzed families. Multiple sequence alignments of the families were generated using PCMA program [41] followed by manual adjustments. All PD-(D/E)XK nuclease structures identified in fold recognition searches were used to derive structure-based alignments in the conserved core regions of the fold encompassing four β-strands and two α-helices. Sequence-to-structure mapping between putative PD-(D/E)XK nuclease families and selected structures in the core region was built manually using consensus alignment approach and 3D assessment [42] based on the results of Meta-BASIC, 3D-Jury and secondary structure predictions (mainly with PSI-PRED) as well as conservation of critical active site residues and hydrophobic patterns.

### Domain architecture and genomic analysis

To detect other conserved protein domains in identified putative restriction endonuclease-like families, their sequences were analyzed with CDD [25] and SMART [43] This analysis also included searches for transmembrane segments (with TMHMM2 [44]), signal peptides (SignalP [45]), low compositional complexity (CEG [46]) and coiled coil (Coils2 [24]) regions as well as internal repeats (Prospero [47]). Regions with no significant sequence similarity to known protein domains were submitted to Meta-BASIC and then to the Meta Server coupled with 3D-Jury system. All identified domains were checked for the conservation of essential elements, including the presence of domain-specific residues. Genomic analysis (neighborhood and/or gene fusion) was performed with STRING [48] to detect possible functional associations.

## Authors' contributions

LK and LNK performed the analyses and drafted the manuscript. LR developed GRDB system. NVG provided thoughtful insights and participated in drafting the manuscript. KG designed and coordinated the study as well as critically edited the manuscript. All authors read and approved the final manuscript.
